# Supplementary material for: Response of soil pH to biochar application in farmland across China: a meta-analysis
Source: PeerJ. 2025 Apr 28;13:e19400. doi: 10.7717/peerj.19400 (PMC12045268; doi:10.7717/peerj.19400)
Supplement: Supplemental Information 3 [file peerj-13-19400-s003.docx]

| **Section and Topic** | **Item #** | **Checklist item** | **Location where item is reported** |
| --- | --- | --- | --- |
| **TITLE** | | |  |
| Title | 1 | The report is identified as a meta-analysis | 1 |
| **ABSTRACT** | | |  |
| Abstract | 2 | See the PRISMA 2020 for Abstracts checklist. | 1 |
| **INTRODUCTION** | | |  |
| Rationale | 3 | Irrespective of the type of biochar applied, it has the potential to raise the pH level of acid soils. However, different types of biochar exhibit divergent effects on soil alkalinity | 1 |
| Objectives | 4 | Biochar, an alkaline material derived from agricultural and forestry waste, can ameliorate soil quality by adjusting soil pH. However, various types of biochar have distinct effects on soil pH due to diversity in feedstock type, pyrolysis temperature, and application rate | 1 |
| **METHODS** | | |  |
| Eligibility criteria | 5 | Based on their original pH, soils were divided into three groups: acid (pH < 6.5), neutral (pH 6.5 = 7.5), and alkaline (pH > 7.5). Biochar feedstocks were also classified into three types: crop straw (e.g., wheat husk, rice straw, corn cob), woody matter (e.g., timbers, leaves, branches), and others (e.g., sewage sludge, livestock and poultry manure, municipal solid waste). Biomass pyrolysis temperatures were ranked into three levels: low (200–400°C), medium (400–600°C), and high (600–800°C). Biochar application rates were grouped into four levels: low (<1%), medium (1%–5%), high (5%–10%), and extremely high (10%–20%). | 3 |
| Information sources | 6 | The Web of Science (https://www.webofscience.com/) and CNKI (https://www.cnki.net/) databases were systematically searched for relevant studies published from January 2010 to July 2022. | 3 |
| Search strategy | 7 | The selection criteria were as follows: 1) the object of the study was farmland soil in China; 2) the design of the study was field experiment, plot experiment, or pot experiment; 3) the experiment consisted of at least one treatment with biochar and one treatment without biochar (control), and other field conditions were consistent; 4) the basic physicochemical properties of soil and biochar samples were reported; and 5) each treatment was replicated at least three times. | 3 |
| Selection process | 8 | 1 reviewer screened each record and each report retrieved | 3 |
| Data collection process | 9 | In addition to directly extracting tabular data, we digitized figure data using GetData version 2.25 (Getdata Pty Ltd, USA). | 3 |
| Data items | 10a | Soil depth, test method (field test or indoor test), initial soil pH, raw material for biochar, preparation temperature, and amount of addition were collected; We recorded the observed data of the response variable—soil pH—in each article, including the mean and standard deviation (SD) or standard error (SE) of control and biochar treatments. | 3 |
|  | 10b | Not | 3 |
| Study risk of bias assessment | 11 | One-way analysis of variance with Tukey's test was used to determine whether there were significant differences in soil pH among different groups or levels of categorical variables. | 3 |
| Effect measures | 12 | In the meta-analysis, the treatments with and without biochar addition were defined as the treatment and control groups, respectively. We computed effect sizes as natural log-transformed response ratios (lnR) | 3 |
| Synthesis methods | 13a | Describe the processes used to decide which studies were eligible for each synthesis (e.g. tabulating the study intervention characteristics and comparing against the planned groups for each synthesis (item #5)). | 3 |
|  | 13b | If SEs were reported in a selected article, they was converted to SD . For articles that did not report SDs or SEs, SDs were estimated to be 10% of the means | 3 |
|  | 13c | Statistical analyses were conducted using OriginPro version 2022 (OriginLab Corp., Northampton, MA, USA). | 4 |
|  | 13d | We conducted weighted meta-analysis, where the mean effect size of each categorical variable was calculated using a fixed effects model. Groups with less than two treatments were omitted from the analysis. Metawin version 2.0 (Rosenberg et al. 2000) was adopted to calculate the overall mean effect size and 95% confidence interval (CI) for each categorical variable. | 4 |
|  | 13e | subgroup analysis | 4 |
|  | 13f | Not | 4 |
| Reporting bias assessment | 14 | get rid of it | 4 |
| Certainty assessment | 15 | If the 95% CI did not overlap with zero, the mean effect size was considered significant; Xt and Xc were considered significantly different if the 95% CI did not overlap from one another. | 4 |
| **RESULTS** | | |  |
| Study selection | 16a | 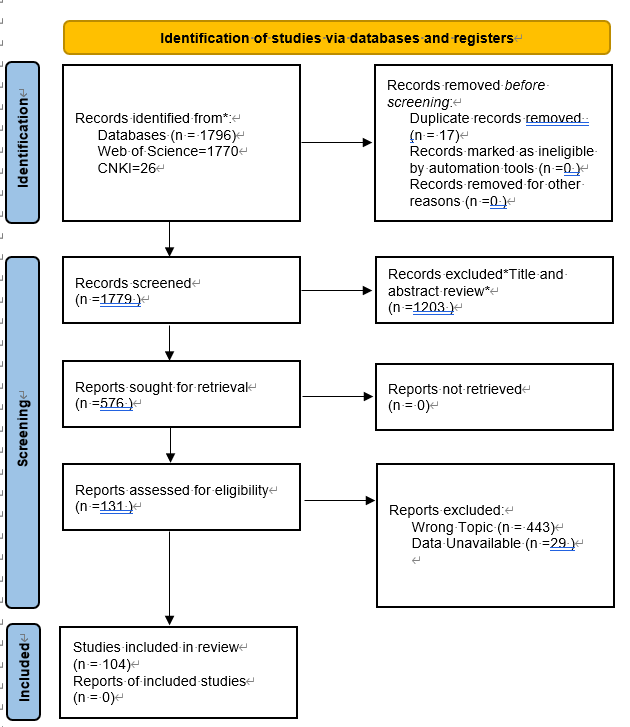 | Not |
|  | 16b | Data Unavailable (n =29 ) | Not |
| Study characteristics | 17 | Not | Not |
| Risk of bias in studies | 18 |  | Not |
| Results of individual studies | 19 | 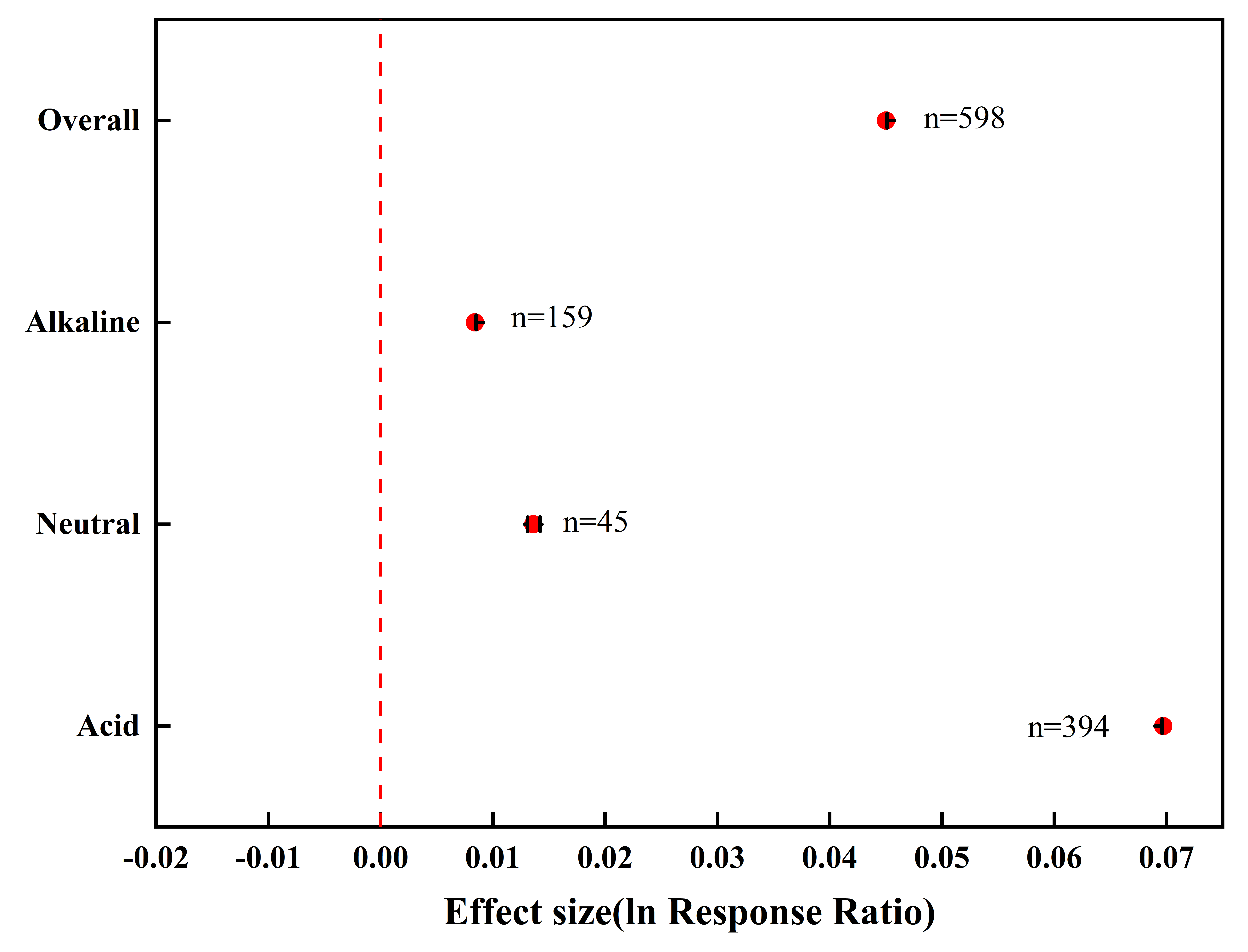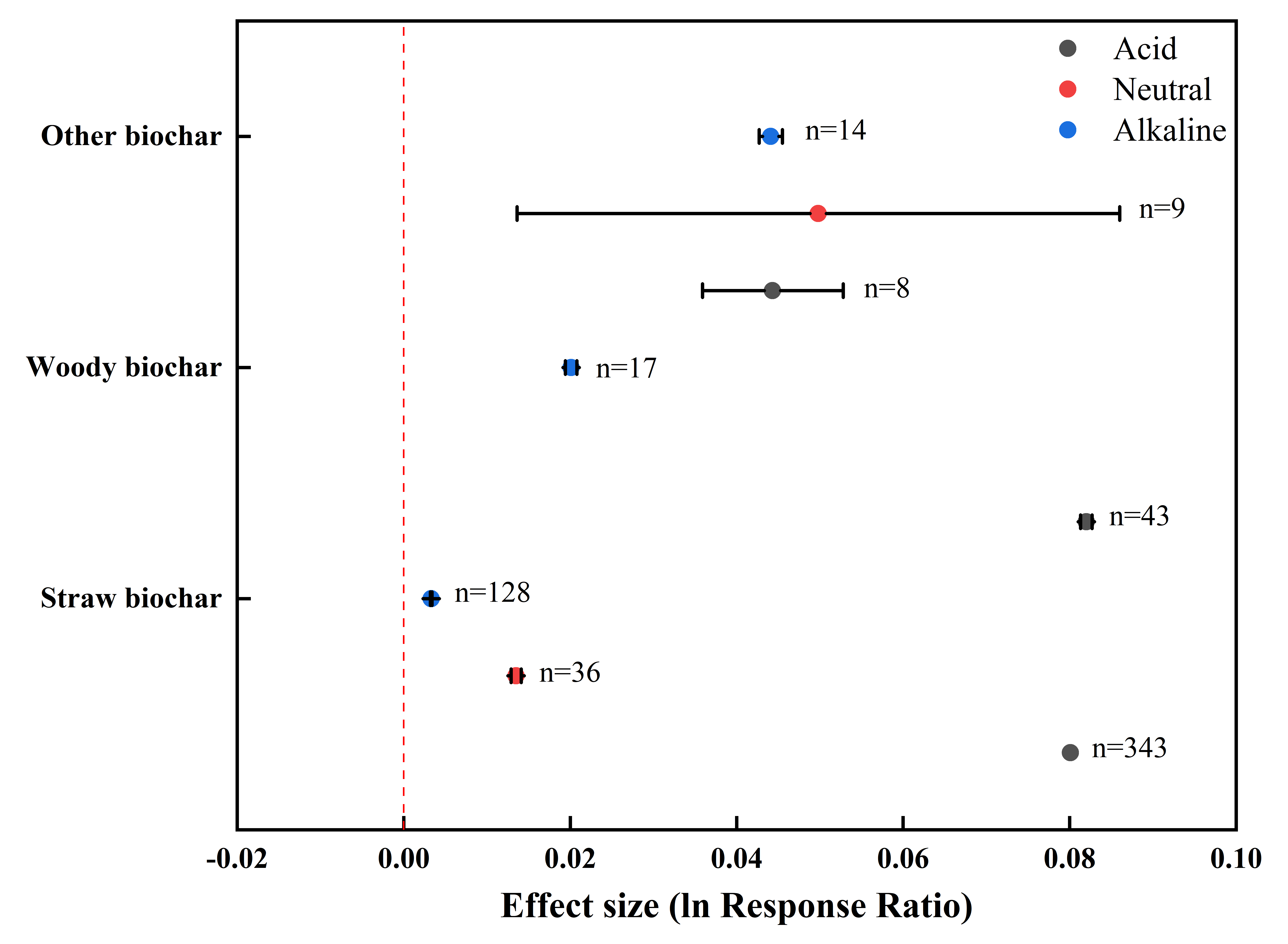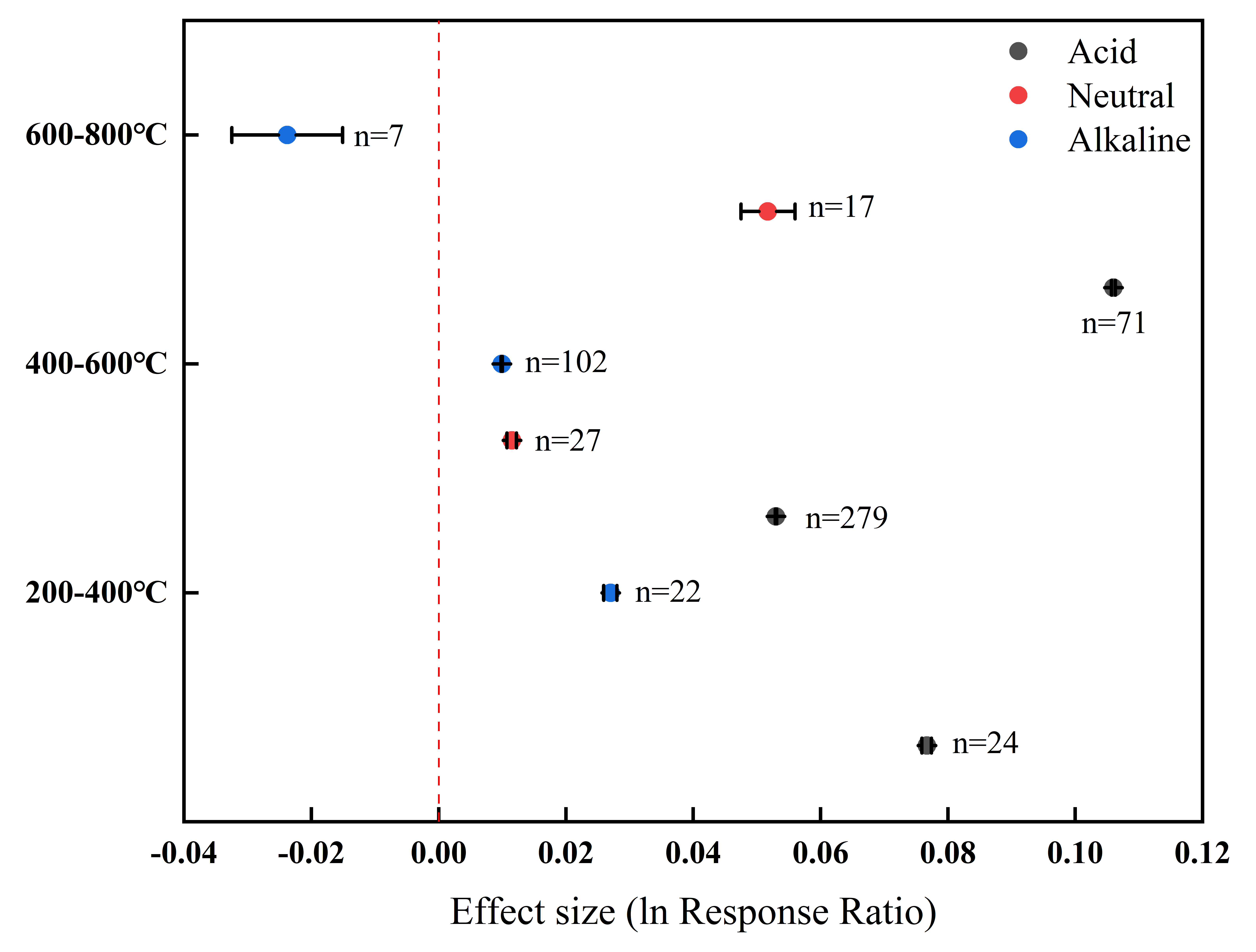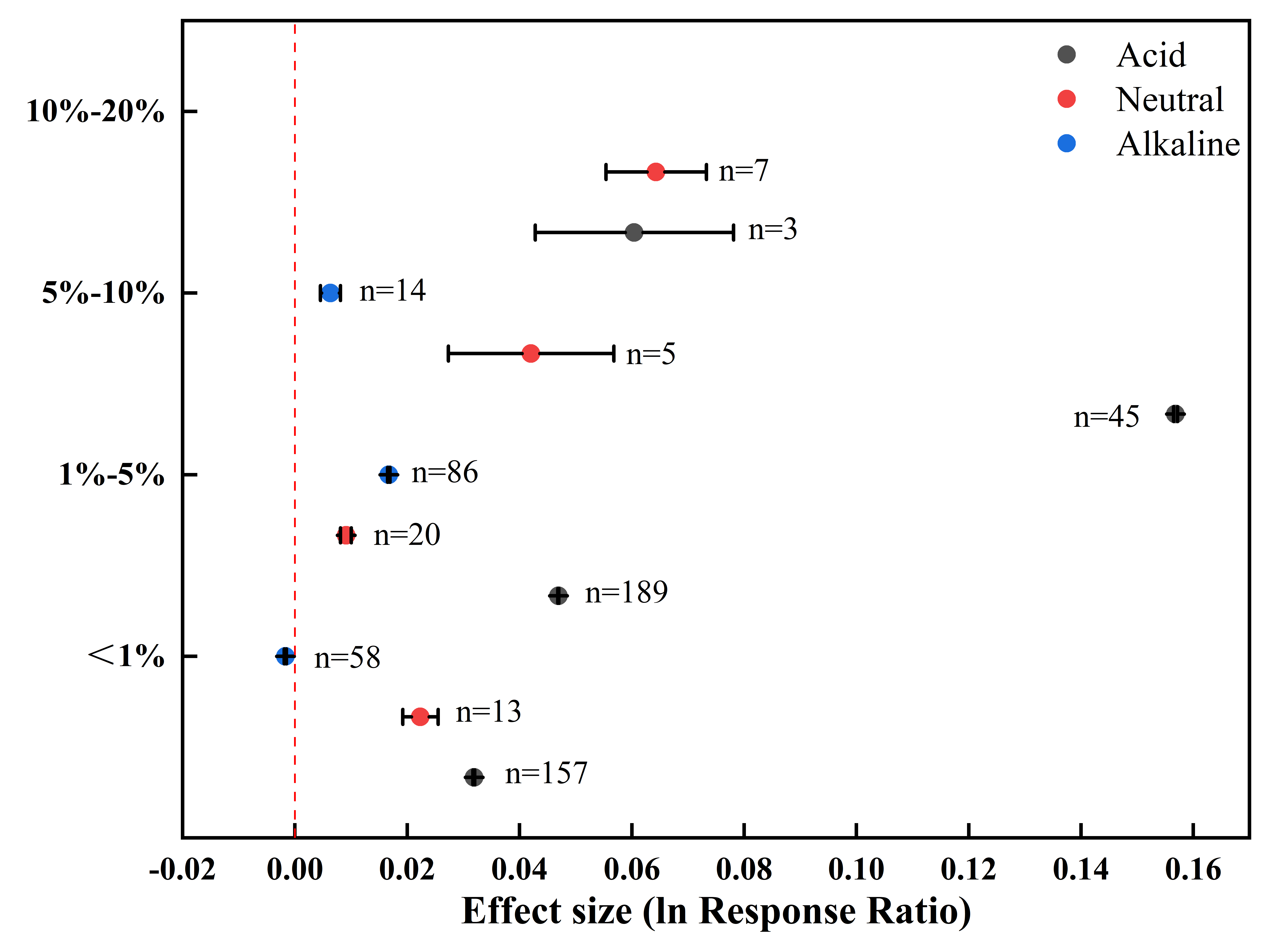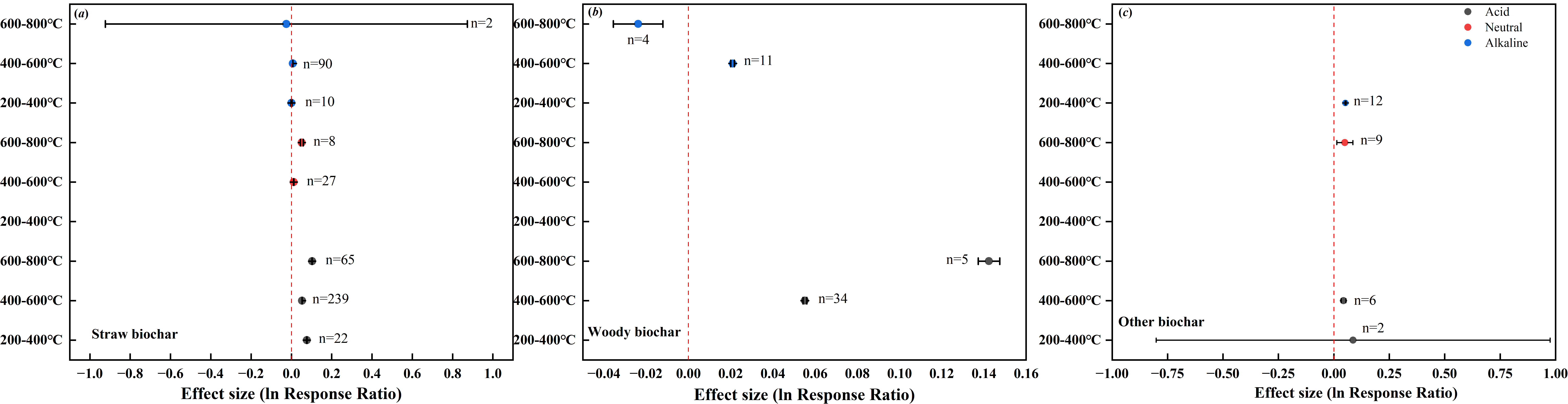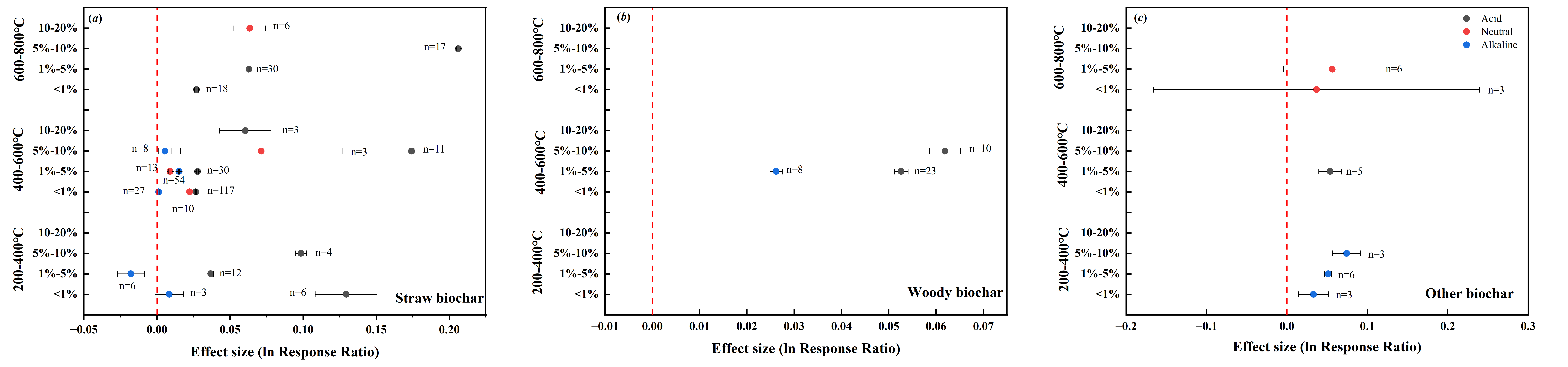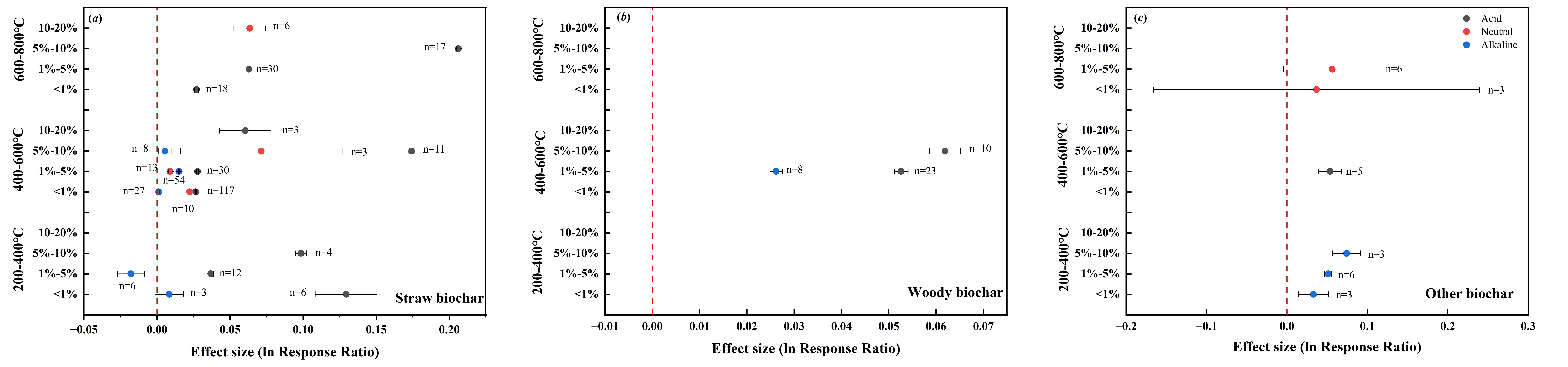 | Not |
| Results of syntheses | 20a | Averaged across the entire dataset, biochar addition significantly increased soil pH in the samples, with an effect value of 0.0450 | Not |
|  | 20b | **Total effect of biochar on soil pH**  Averaged across the entire dataset, biochar addition significantly increased soil pH in the samples, with an effect value of 0.0450 (Fig. 1). When separating the samples into acidic, neutral, and alkaline soils, biochar exhibited a positive effect on the pH of acidic soils (0.0662), and this effect was greater than that for neutral (0.0136) and alkaline soils (0.0054).  **Effect of biochar from different feedstocks on soil pH**  The addition of biochar derived from various feedstocks displayed a positive effect on soil pH (Fig. 2). The response of soil pH to straw biochar was greater in acid soils (0.0801) than in neutral and alkaline soils (0.0135 and 0.0033, respectively). Woody biochar exhibited a similar effect to straw biochar in both acid and alkaline soils. However, other biochar displayed a greater effect in neutral soils, with no significant difference between acid and alkaline soils. Among the three feedstock types, woody biochar and straw biochar had prominent effects on acid soils (0.0820 and 0.0801, respectively), which accounted for a significant increase in soil pH. On the contrary, other biochar had the greatest effect on neutral (0.0498) and alkaline (0.0441) soils.  **Effect of biochar with different pyrolysis temperatures on soil pH**  Upon addition of biochar prepared at 200–400°C, the pH of both acid and alkaline soils responded positively, and the effect on acid soils was greater than that on alkaline soils (Fig. 3). The addition of biochar prepared at 400–600°C exhibited a positive effect on soil pH in all three groups, and the greatest effect emerged in acid soils. Notably, adding the biochar prepared at 600–800°C exerted a negative effect on the pH of alkaline soils, which contrasted with its positive effect on the pH of neutral and acid soils. Among the three temperature levels, the positive effect of high-temperature biochar was the most remarkable on acid (0.1008) and neutral (0.0517) soils. However, for alkaline soils, the greatest negative effect was attributed to high-temperature biochar (–0.014), whereas the most evident positive effect was posed by low-temperature biochar (0.0270).  **Effect of biochar applied at different rates on soil pH**  The effects of different biochar application rates on soil pH were divergent (Fig. 4). Biochar addition at low rates <1% mainly positively affected the pH of acid (0.0319) and neutral (0.0223) soils, with a minimal negative effect on alkaline soils (–0.0017). The addition of 1%–5% and 5%–10% biochar had an exclusively positive effect on soil pH over a range of acidity, neutrality, and alkalinity. The positive effect of biochar was generally enhanced with increasing application rate, and adding 10%–20% biochar specifically affected the pH of acid and neutral soils. Among the three levels of application rate, 5%–10% biochar exhibited the greatest positive effect on acid soils (0.1568), whereas 10%–20% and 1%–5% biochar respectively showed the most prominent effect on neutral (0.0643) and alkaline (0.0167) soils.  **Interaction effects of biochar feedstock and pyrolysis temperature on soil pH**  Biochar feedstock and pyrolysis temperature showed varied interaction effects on soil pH (Fig. 5). The responses of pH in acid and neutral soils to straw biochar with different pyrolysis temperatures (Fig. 5a) were consistent with the response patterns to all biochar (Fig. 3). In the case of alkaline soils, straw biochar prepared at 200–400°C and 400–600°C had a slight positive effect on soil pH (0.0003 and 0.0075, respectively), whereas a negative effect was observed for straw biochar prepared at 600–800°C (–0.0250).  The positive effect of woody biochar on the pH of acid soils was heightened with increasing pyrolysis temperature. In alkaline soils, woody biochar showed contrasting effects on soil pH depending on pyrolysis temperature, as indicated by a positive effect value at 400–600°C (0.0210) and a negative effect value at 600–800°C (–0.0238; Fig. 5b). The effects of other biochar prepared at different temperatures were positive across all soil groups (Fig. 5c).  **Interaction effect of biochar feedstock and application rate on soil pH**  There were distinct interaction effects of biochar feedstock and application rate on soil pH (Fig. 6) Specifically, different straw biochar rates had a positive effect on the pH of acid and neutral soils (Fig. 6a), and the response of pH in acid soils was consistent with the pattern observed for all biochar (Fig. 4). In the case of alkaline soils, adding 1%–5% straw biochar had a small positive effect (0.0105) on soil pH, whereas lower and higher straw biochar rates had a minor negative effect on soil pH (–0.0022 and –0.0019, respectively; Fig. 6a).  The response of pH in acid soils to different woody biochar rates was also consistent with the pattern observed for all biochar (Fig. 4). In alkaline soils, the effect of woody biochar rate on soil pH resembled that of straw biochar rate. When woody biochar was added at rates of 1%–5%, there was a positive effect on soil pH (0.0263). Conversely, a negative effect emerged with woody biochar addition at lower and higher rates (–0.0001 and –0.0259, respectively; Fig. 6b).  The addition of other biochar at different rates had consistent positive effects on the pH of acid, neutral, and alkaline soils (Fig. 6c). Together, the results indicate that greatest positive effect on soil pH was observed in acid and neutral soils upon straw biochar addition at high (5%–10%) and extremely high (10%–20%) rates, respectively. The greatest negative effect observed in alkaline soils was attributed to woody biochar addition at high rates.  **Interaction effect of biochar feedstock, pyrolysis temperature, and application rate on soil pH**  Regardless of the application rate, the addition of straw biochar prepared at 200–400°C exhibited a positive effect on the pH of acid soils (0.0767). However, when this biochar was applied between rates of 1%–5%, a negative effect on soil pH emerged in alkaline soils (–0.0178). The effect of straw biochar prepared at 600–800°C was positive across all soils, being the strongest in acid soils at the application rate of 5%–10%. Woody biochar prepared at 400–600°C also positively affected the pH of both acid and alkaline soils, and this effect was strengthened in acid soils with increasing application rate. The addition of other biochar with different pyrolysis temperatures had a positive effect on the pH of acid, neutral, and alkaline soils, and there was an upward trend in this effect with increasing application rate (Fig. 7c).  Among the different treatments, the pH of acid soils was positively affected the most by addition of 5%–10% straw biochar with high temperatures (600–800°C; 0.2062). The same application rate of medium-temperature straw biochar (400–600°C) was most effective in increasing the pH of neutral soils (0.0713). In alkaline soils, however, the greatest positive response of pH was observed upon addition of 5%–10% other biochar prepared at 200–400°C (0.0742), whereas the addition of 1%–5% straw biochar prepared at 200–400°C had a negative effect on alkaline soils (-0.0178). | 4-6 |
|  | 20c | Not | Not |
|  | 20d | Not | Not |
| Reporting biases | 21 | Not | Not |
| Certainty of evidence | 22 | Not | Not |
| **DISCUSSION** | | |  |
| Discussion | 23a | Provide a general interpretation of the results in the context of other evidence. | 5-8 |
|  | 23b | Large-scale application of biochar, a promising soil amendment, is plagued by divergent consequences in different soils. In this meta-analysis, we demonstrated the benefits of biochar addition in ameliorating the pH of acid, neutral, and alkaline soils in farmland systems across China based on experimental data published over the past 12 years (Fig. 1). More importantly, we linked biochar-induced changes in soil pH to feedstock type, pyrolysis temperature, and application rate (Figs. 2–4). The findings present a holistic picture of how soil pH responds to various types of biochar in farmland systems, which has practical implications for the development of guidelines on biochar application.  Our results corroborated earlier research (Shi et al. 2019) that the addition of biochar with various feedstocks, pyrolysis temperatures, and application rates notably increased the pH of acid farmland soils (Figs. 2–7). This positive effect on soil pH is attributable to the alkaline nature of biochar. During pyrolysis, the acid functional groups and cations present in the feedstock are incorporated to form alkaline substances, such as –COO–, –O–, carbonates, and oxides. After biochar application, these alkaline substances play a role in alleviating soil acidity by neutralizing H+ ions and reacting with Al3+ ions in the soil (Dai et al. 2017). Fuertes et al. (2010) showed that 1 ton of hydrochar could have a limiting effect of 39.6 kg CaCO3. In addition to H+ and Al3+ ion exchange, biochar can elevate soil pH through the input of cations, such as Ca2+, K+, and Mg2+ (Glaser et al. 2002). Therefore, it is not surprising that the pH of acidic soils increases in response to biochar addition.  We additionally found that with regard to the feedstock used, biochar addition consistently increased the pH of farmland soils. In acid soils, woody biochar exhibited a greater effect than straw biochar and other biochar, whereas in neutral and alkaline soils, other biochar exerted the greatest effect on soil pH (Fig. 2). According to Wijitkosum. (2022) and Schmidt &Wilson (2014), biochar made from woody matter has low pH as a result of low ash content. | 5 |
|  | 23c | As such, woody biochar may not be as effective in increasing the pH of acid soils as biochar derived from crop straw and other materials. This contradicts our conclusion based on the meta-analysis. One possible reason for this contradiction is that in our dataset, the pH ranges of straw biochar (6.48–11.32) and other biochar (7.97–8.80) collected from acid soils are lower than that of woody biochar (7.90–11.30). Therefore, woody biochar has the best performance in increasing the pH of acid soils. In alkaline soils, the pH of other biochar is generally higher than soil pH, whereas woody biochar and straw biochar sometimes have lower pH values lower than the soil. This explains why other biochar has an overall stronger positive effect on the pH of alkaline soils. The situation of neutral soils is similar to that of alkaline soils. | 5 |
|  | 23d | The results reported in this study showed that the addition of biochar with different feedstocks and pyrolysis temperatures had similar effects on the pH of acid and neutral soils, which contrasted with the response of pH in alkaline soils (Figs. 3, 5). In principle, the higher the pyrolysis temperature, the higher the biochar pH (Geng et al. 2022). However, the pH of alkaline soils responded differently to woody biochar and straw biochar, with higher pyrolysis temperatures contributing less to soil pH. This is because based on our data, the soils treated with biochar prepared at higher temperatures had higher initial pH values. The responses of soil pH to different application rates of woody biochar and other biochar were inconsistent with the response patterns to the application rates of total biochar in alkaline soils. This was also due to the minimal difference between biochar pH and soil pH in our dataset. | 5 |
| **OTHER INFORMATION** | | |  |
| Registration and protocol | 24a | The review was not registered. | Not |
|  | 24b | A protocol was not prepared. | Not |
|  | 24c | Not | Not |
| Support | 25 | Not | Not |
| Competing interests | 26 | The authors declare no conflicts of interest. | Not |
| Availability of data, code and other materials | 27 | Report which of the following are publicly available and where they can be found: data extracted from included studies; data used for all analyses; analytic code | Not |

*From:*  Page MJ, McKenzie JE, Bossuyt PM, Boutron I, Hoffmann TC, Mulrow CD, et al. The PRISMA 2020 statement: an updated guideline for reporting systematic reviews. BMJ 2021;372:n71. doi: 10.1136/bmj.n71
