# Supplementary material for: Response of soil pH to biochar application in farmland across China: a meta-analysis
Source: PeerJ. 2025 Apr 28;13:e19400. doi: 10.7717/peerj.19400 (PMC12045268; doi:10.7717/peerj.19400)
Supplement: Supplemental Information 4 [file peerj-13-19400-s004.docx]

**Identification of studies via databases and registers**

Records removed *before screening*:

Duplicate records removed (n = 17)

Records marked as ineligible by automation tools (n =0 )

Records removed for other reasons (n =0 )

Records identified from*:

Databases (n = 1796)

Web of Science=1770

CNKI=26

**Identification**

Records screened

(n =1779 )

Records excluded*Title and abstract review*

(n =1203 )

Reports sought for retrieval

(n =576 )

Reports not retrieved

(n = 0)

**Screening**

Reports assessed for eligibility

(n =131 )

Reports excluded:

Wrong Topic (n = 443)

Data Unavailable (n =29 )

Studies included in review

(n = 104)

Reports of included studies

(n = 0)

**Included**

*Consider, if feasible to do so, reporting the number of records identified from each database or register searched (rather than the total number across all databases/registers).

**If automation tools were used, indicate how many records were excluded by a human and how many were excluded by automation tools.

Source: Page MJ, et al. BMJ 2021;372:n71. doi: 10.1136/bmj.n71.

This work is licensed under CC BY 4.0. To view a copy of this license, visit <https://creativecommons.org/licenses/by/4.0/>
