# Supplementary material for: Response of soil pH to biochar application in farmland across China: a meta-analysis
Source: PeerJ. 2025 Apr 28;13:e19400. doi: 10.7717/peerj.19400 (PMC12045268; doi:10.7717/peerj.19400)
Supplement: Supplemental Information 5 [file peerj-13-19400-s005.docx]

The reason for this meta-analysis was that various types of biochar have distinct effects on soil pH due to diversity in feedstock type, pyrolysis temperature, and application rate.

Despite some meta-analyses revealing biochar-induced changes in soil physicochemical properties , there is still a paucity of research deciphering the responses of soil pH to various types of biochar in farmland systems across large spatial scales. To fill this knowledge gap, we compared the effects of biochar with different feedstock types, pyrolysis temperatures, and application rates on the pH of acid, neutral, alkaline soils in China through a meta-analysis of published articles. Results of this study could provide mechanistic insights into the divergent role of biochar in improving soil quality from a pH perspective.
